# Uniqueness of a nonlinear integro-differential equation with nonlocal boundary condition and variable coefficients

**DOI:** 10.1186/s13661-023-01713-6

**Published:** 2023-03-15

**Authors:** Chenkuan Li

**Affiliations:** Department of Mathematics and Computer Science, Brandon University, Brandon, Manitoba R7A 6A9 Canada

**Keywords:** 34B15, 34A12, 26A33, Liouville-Caputo integro-differential equation, Banach’s contractive principle, Mittag-Leffler function, Babenko’s approach

## Abstract

This paper studies the uniqueness of solutions to a two-term nonlinear fractional integro-differential equation with nonlocal boundary condition and variable coefficients based on the Mittag-Leffler function, Babenko’s approach, and Banach’s contractive principle. An example is also provided to illustrate the applications of our theorem.

## Introduction

Let $T > 0$. The Riemann-Liouville fractional integral $I^{\alpha}$ of order $\alpha \in R^{+}$ is defined for function $u(x)$ as (see [1, 2])

$$ \bigl(I^{\alpha }u\bigr) (x) = \frac{1}{\Gamma (\alpha )} \int _{0}^{x} (x - t)^{ \alpha - 1} u(t) \,d t, \quad x \in [0, T]. $$

In particular,

$$ \bigl(I^{0} u\bigr) (x) = u(x), $$

from [3].

Let $l \in N = \{1, 2, 3, \ldots \}$. The Liouville-Caputo derivative of fractional order $\alpha \in R^{+}$ of function $u(x)$ is defined as

$$ \bigl({}_{C} D^{\alpha }u\bigr) (x) = I^{l - \alpha} \frac{d^{l}}{d x^{l}} u(x) = \frac{1}{\Gamma (l - \alpha )} \int _{0}^{x} (x - t)^{l - \alpha - 1} u^{(l)}(t) \,d t, $$

where $l - 1 < \alpha \leq l$.

Let $a(x) \in C[0, T]$, $g : [0, T] \times R \rightarrow R$ and $f : C[0, T] \rightarrow R$. We shall study the uniqueness of solutions for the following nonlinear integro-differential equation with nonlocal boundary condition and variable coefficients for $l < \alpha \leq l + 1$:

1.1 $$\begin{aligned} \textstyle\begin{cases} {}_{C} D^{\alpha }u(x) + a(x) I^{\beta} u(x) = g(x, u(x)), \quad x \in [0, T], \\ u(0) = - f(u), \qquad u''(0) = \cdots = u^{(l )}(0) = 0, \\ \int _{0}^{T} u(x) \,d x = \lambda , \end{cases}\displaystyle \end{aligned}$$

where *λ* is a constant. In particular, for $l = 1$, equation (1.1) turns out to be

1.2 $$\begin{aligned} \textstyle\begin{cases} {}_{C} D^{\alpha }u(x) + a(x) I^{\beta} u(x) = g(x, u(x)), \quad x \in [0, T], \\ u(0) = - f(u), \qquad \int _{0}^{T} u(x) \,d x = \lambda . \end{cases}\displaystyle \end{aligned}$$

Boundary value problems of fractional differential equations have recently attracted many researchers and emerged as an important field of research due to their applications in various areas of science and engineering, such as control theory, wave propagation, mechanics, and biology [4–21]. In 2014, Tariboon et al. [4] investigated the existence and uniqueness of solutions for the following fractional differential equation:

$$ {}_{C} D^{\alpha }u(x) = g\bigl(x, u(x)\bigr), \quad 1 < \alpha \leq 2, x \in [0, T], $$

subject to nonlocal fractional integral boundary conditions:

$$ \sum_{i = 1}^{m} \lambda _{i} u( \eta _{i}) = \omega _{1}, \qquad \sum _{j = 1}^{n} \mu _{j} \bigl( I^{\beta _{j}} u(T) - I^{\beta _{j}} u(\zeta _{j}) \bigr) = \omega _{2}, $$

where $g : [0, T] \times R \rightarrow R$ is a continuous function, $\lambda _{i}, \mu _{j} \in R$ for all $i = 1, 2, \ldots , m$, $j = 1, 2, \ldots , n$, and $\omega _{1}, \omega _{2} \in R$, using Krasnoselskii’s fixed point theorem, Banach’s contractive principle and Leray-Schauder’s nonlinear alternative.

In particular, for all $\alpha _{i} = \beta _{j} = 1$, the above boundary conditions become

$$\begin{aligned}& \lambda _{1} \int _{0}^{\eta _{1}} u(x) \,d x + \cdots + \lambda _{m} \int _{0}^{\eta _{m}} u(x) \,d x = \omega _{1}, \\& \mu _{1} \int _{\zeta _{1}}^{T} u(x) \,d x + \cdots + \mu _{n} \int _{ \zeta _{n}}^{T} u(x) \,d x = \omega _{2}. \end{aligned}$$

In 2013, Yan et al. [5] studied the existence and uniqueness of solutions for the following boundary value problem of fractional differential equation based on several standard fixed point theorems:

1.3 $$ {}_{C} D^{\alpha }u(x) = g\bigl(x, u(x)\bigr), \quad 1 < \alpha \leq 2, x \in [0, T], $$

with the nonlocal boundary conditions

$$ u(0) = g(u), \qquad \int _{0}^{T} u(x) \,d x = m, $$

where $g : C^{2}[0, T] \rightarrow R$ is a $C^{2}$ continuous functional. Clearly, equation (1.3) with its nonlocal boundary conditions is a special case of equation (1.2) by setting $a(x) = 0$. Very recently, Li et al. [22] studied the uniqueness and existence for the following nonlinear integro-differential equation with the boundary condition using several fixed point theorems:

$$\begin{aligned} \textstyle\begin{cases} {}_{C} D_{p}^{\alpha }u(x) + \mu I_{p}^{\beta} u(x) = g(x, u(x)), \quad x \in [p, q], l - 1 < \alpha \leq l, \beta \geq 0, \\ u(p) = u'(p) = \cdots = u^{(l -2 )}(p) = 0 = u^{(l -1 )}(q), \end{cases}\displaystyle \end{aligned}$$

where *μ* is a constant, and $0 \leq p < q < + \infty $.

Very little is known in modern literature about boundary value problems of fractional integro-differential equations with integral boundary conditions and variable coefficients. We will work on equation (1.1), which, to the best of the author’s knowledge, is new and requires the following preliminaries.

We define the Banach space $C[0, T]$ with the norm

$$ \lVert u \rVert = \max_{x \in [0, T]} \bigl\vert u(x) \bigr\vert < + \infty . $$

The two-parameter Mittag-Leffler function [2] is defined by

$$ E_{\alpha , \beta}(z) = \sum_{k = 0}^{\infty } \frac{z^{k}}{\Gamma (\alpha k + \beta )},\quad z \in C, \alpha , \beta > 0. $$

Babenko’s approach [23] is a powerful tool for solving differential and integro-differential equations with initial conditions by treating bounded integral operators as normal variables. The method itself is similar to the Laplace transform while working on differential and integral equations with constant coefficients, but it can be applied to equations with continuous and bounded variable coefficients. As an example to demonstrate the technique, we will show the following lemma, which plays an essential role in deriving our main theorem.

### Lemma 1

*Let* $a, h : [0, T] \rightarrow R$ *be continuous functions and* $f : C[0, T] \rightarrow R$ *be a continuous functional*. *Assume that*

$$ \frac{2M T^{\alpha + \beta}}{\Gamma (\alpha + \beta + 2)} E_{\alpha + \beta , 1} \bigl( M T^{\alpha + \beta} \bigr)< 1, $$

*where* *M* *is a constant satisfying*

$$ \lVert a \rVert = \max_{x \in [0, T]} \bigl\vert a(x) \bigr\vert \leq M. $$

*Then* $u(x)$ *is a solution to the following nonlinear integro*-*differential equation*

1.4 $$\begin{aligned} \textstyle\begin{cases} {}_{C} D^{\alpha }u(x) + a(x) I^{\beta} u(x) = h(x), \quad l < \alpha \leq l + 1, x \in [0, T], \\ u(0) = - f(u), \qquad u''(0) = \cdots = u^{(l )}(0) = 0, \\ \int _{0}^{T} u(x) \,d x = \lambda , \end{cases}\displaystyle \end{aligned}$$

*if and only if* $u(x)$ *satisfies the integral equation*

$$\begin{aligned} u(x) = & \sum_{j = 0}^{\infty }(-1)^{j} \bigl(I^{\alpha }a(x) I^{ \beta} \bigr)^{j} I^{\alpha }h(x) - f(u) \sum_{j = 0}^{\infty }(-1)^{j} \bigl(I^{\alpha }a(x) I^{\beta} \bigr)^{j} \cdot 1 \\ &{}- \frac{2}{\Gamma (\alpha + 1) T^{2}} \int _{0}^{T} ( T - t)^{ \alpha }h(t) \,dt \sum _{j = 0}^{\infty }(-1)^{j} \bigl(I^{\alpha }a(x) I^{\beta} \bigr)^{j} x \\ &{}+ \frac{2}{\Gamma (\alpha + 1) \Gamma (\beta ) T^{2} } \int _{0}^{T} (T - x_{1})^{\alpha }a (x_{1}) \,d x_{1} \int _{0}^{x_{1}} (x_{1} - t)^{ \beta - 1} u(t) \,d t \\ &{}\cdot \sum_{j = 0}^{\infty }(-1)^{j} \bigl(I^{\alpha }a(x) I^{\beta} \bigr)^{j} x + \frac{2 \lambda }{T^{2}} \sum_{j = 0}^{\infty }(-1)^{j} \bigl(I^{\alpha }a(x) I^{\beta} \bigr)^{j} x \\ &{}+ \frac{2 f(u)}{T} \sum_{j = 0}^{\infty }(-1)^{j} \bigl(I^{ \alpha }a(x) I^{\beta} \bigr)^{j} x, \end{aligned}$$

*in the space* $C[0, T]$.

### Proof

Clearly,

$$ I^{\alpha }\bigl({}_{C} D^{\alpha}\bigr) u(x) = u(x) + f(u) + c_{1} x, $$

using

$$ u(0) = -f(u), \qquad u''(0) = \cdots = u^{(l )}(0) = 0. $$

Hence, applying the operator $I^{\alpha}$ to both sides of the equation

$$ {}_{C} D^{\alpha }u(x) + a(x) I^{\beta} u(x) = h(x), $$

we get

$$ u(x) + f(u) + c_{1} x + I^{\alpha }a(x) I^{\beta} u(x) = I^{ \alpha }h(x). $$

Obviously,

$$\begin{aligned} \int _{0}^{T} I^{\alpha }h(x) \,d x =& \frac{1}{\Gamma (\alpha )} \int _{0}^{T} \,d x \int _{0}^{x} (x - t)^{\alpha - 1} h(t) \,d t = \frac{1}{\Gamma (\alpha )} \int _{0}^{T} h(t) \,dt \int _{t}^{T} (x - t)^{ \alpha - 1} \,dx \\ =& \frac{1}{\Gamma (\alpha + 1)} \int _{0}^{T} ( T - t)^{\alpha }h(t) \,dt. \end{aligned}$$

Similarly,

$$ \int _{0}^{T} I^{\alpha }a(x) I^{\beta} u(x) \,d x = \frac{1}{\Gamma (\alpha + 1) \Gamma (\beta )} \int _{0}^{T} (T - x_{1})^{ \alpha }a (x_{1}) \,d x_{1} \int _{0}^{x_{1}} (x_{1} - t)^{\beta - 1} u(t) \,d t. $$

Thus,

$$ \int _{0}^{T} u(x) \,d x + \int _{0}^{T} f(u) \,dx + c_{1} \int _{0}^{T} x \,d x + \int _{0}^{T} I^{\alpha }a(x) I^{\beta} u(x) \,d x = \int _{0}^{T} I^{\alpha }h(x) \,dx, $$

which implies that

$$ \lambda + f(u) T + \frac{T^{2}}{2} c_{1} = \int _{0}^{T} I^{\alpha }h(x) \,dx - \int _{0}^{T} I^{\alpha }a(x) I^{\beta} u(x) \,d x, $$

by noting that $f(u) \in R$. So,

$$\begin{aligned} c_{1} = & \frac{2}{\Gamma (\alpha + 1) T^{2}} \int _{0}^{T} ( T - t)^{ \alpha }h(t) \,dt \\ &{}- \frac{2}{\Gamma (\alpha + 1) \Gamma (\beta ) T^{2} } \int _{0}^{T} (T - x_{1})^{\alpha }a (x_{1}) \,d x_{1} \int _{0}^{x_{1}} (x_{1} - t)^{ \beta - 1} u(t) \,d t \\ &{}- \frac{2 \lambda}{T^{2}} - \frac{2 f(u)}{T}, \end{aligned}$$

and

$$ \bigl(1 + I^{\alpha }a(x) I^{\beta} \bigr) u(x) = I^{\alpha }h(x) - f(u) - c_{1} x. $$

Treating the factor $(1 + I^{\alpha }a(x) I^{\beta} )$ as a variable, we deduce that by Babenko’s approach

$$\begin{aligned} u(x) = & \bigl(1 + I^{\alpha }a(x) I^{\beta} \bigr)^{-1} \bigl[ I^{\alpha }h(x) - f(u) - c_{1} x \bigr] \\ = & \sum_{j = 0}^{\infty }(-1)^{j} \bigl(I^{\alpha }a(x) I^{ \beta} \bigr)^{j} I^{\alpha }h(x) - f(u) \sum_{j = 0}^{\infty }(-1)^{j} \bigl(I^{\alpha }a(x) I^{\beta} \bigr)^{j} \cdot 1 \\ &{}- c_{1} \sum_{j = 0}^{\infty }(-1)^{j} \bigl(I^{\alpha }a(x) I^{ \beta} \bigr)^{j} x \\ = & \sum_{j = 0}^{\infty }(-1)^{j} \bigl(I^{\alpha }a(x) I^{ \beta} \bigr)^{j} I^{\alpha }h(x) - f(u) \sum_{j = 0}^{\infty }(-1)^{j} \bigl(I^{\alpha }a(x) I^{\beta} \bigr)^{j} \cdot 1 \\ &{}- \frac{2}{\Gamma (\alpha + 1) T^{2}} \int _{0}^{T} ( T - t)^{ \alpha }h(t) \,dt \cdot \sum_{j = 0}^{\infty }(-1)^{j} \bigl(I^{ \alpha }a(x) I^{\beta} \bigr)^{j} x \\ &{}+ \frac{2}{\Gamma (\alpha + 1) \Gamma (\beta ) T^{2} } \int _{0}^{T} (T - x_{1})^{\alpha }a (x_{1}) \,d x_{1} \int _{0}^{x_{1}} (x_{1} - t)^{ \beta - 1} u(t) \,d t \\ &{}\cdot \sum_{j = 0}^{\infty }(-1)^{j} \bigl(I^{\alpha }a(x) I^{\beta} \bigr)^{j} x + \frac{2 \lambda }{T^{2}} \sum_{j = 0}^{\infty }(-1)^{j} \bigl(I^{\alpha }a(x) I^{\beta} \bigr)^{j} x \\ &{}+ \frac{2 f(u)}{T} \sum_{j = 0}^{\infty }(-1)^{j} \bigl(I^{ \alpha }a(x) I^{\beta} \bigr)^{j} x. \end{aligned}$$

It remains to show that $u \in C[0, T]$. Clearly,

$$ \bigl\lVert I^{\alpha }h \bigr\rVert = \frac{1}{\Gamma (\alpha )} \max _{x \in [0, T]} \biggl\vert \int _{0}^{x} (x - t)^{\alpha - 1} h(t) \,d t \biggr\vert \leq \frac{T^{\alpha }}{\Gamma (\alpha + 1)} \lVert h \rVert . $$

Hence, we infer that

1.5 $$\begin{aligned} \lVert u \rVert \leq & \sum_{j = 0}^{\infty } \bigl\lVert \bigl(I^{\alpha }a(x) I^{\beta } \bigr)^{j} I^{\alpha } \bigr\rVert \lVert h \rVert + \bigl\vert f(u) \bigr\vert \sum _{j = 0}^{\infty } \bigl\lVert \bigl(I^{\alpha }a(x) I^{\beta } \bigr)^{j} \bigr\rVert \\ &{}+ \frac{2 \lVert h \rVert }{\Gamma (\alpha + 2) T^{2}} T^{ \alpha + 1} \sum_{j = 0}^{\infty } \bigl\lVert \bigl(I^{\alpha }a(x) I^{\beta } \bigr)^{j} \bigr\rVert \lVert x \rVert \\ &{}+ \frac{2 M \lVert u \rVert T^{\alpha + \beta - 1}}{\Gamma (\alpha + \beta + 2) } \sum_{j = 0}^{\infty } \bigl\lVert \bigl(I^{\alpha }a(x) I^{ \beta } \bigr)^{j} \bigr\rVert \lVert x \rVert \\ &{}+ \frac{2 \vert \lambda \vert }{T^{2}} \sum_{j = 0}^{\infty } \bigl\lVert \bigl(I^{\alpha }a(x) I^{\beta } \bigr)^{j} \bigr\rVert \lVert x \rVert + \frac{2 \vert f(u) \vert }{T} \sum_{j = 0}^{ \infty } \bigl\lVert \bigl(I^{\alpha }a(x) I^{\beta } \bigr)^{j} \bigr\rVert \lVert x \rVert \\ \leq & \lVert h \rVert \sum_{j = 0}^{\infty } \frac{M^{j} T^{(\alpha + \beta ) j + \alpha }}{\Gamma ((\alpha + \beta ) j + \alpha + 1)} + \bigl\vert f(u) \bigr\vert \sum _{j = 0}^{\infty } \frac{M^{j} T^{(\alpha + \beta ) j}}{\Gamma ((\alpha + \beta )j + 1)} \\ &{}+ \frac{2 \lVert h \rVert T^{\alpha }}{\Gamma (\alpha + 2)} \sum_{j = 0}^{\infty } \frac{M^{j} T^{(\alpha + \beta ) j}}{\Gamma ((\alpha + \beta )j + 1)} + \frac{2 M \lVert u \rVert T^{\alpha + \beta}}{\Gamma (\alpha + \beta + 2)} \sum_{j = 0}^{\infty } \frac{M^{j} T^{(\alpha + \beta ) j}}{\Gamma ((\alpha + \beta )j + 1)} \\ &{}+ \biggl[ \frac{2 \vert \lambda \vert }{T} + 2 \bigl\vert f(u) \bigr\vert \biggr] \sum _{j = 0}^{\infty } \frac{M^{j} T^{(\alpha + \beta ) j}}{\Gamma ((\alpha + \beta )j + 1)}. \end{aligned}$$

This implies that

$$\begin{aligned}& \biggl( 1 - \frac{2 M T^{\alpha + \beta}}{\Gamma (\alpha + \beta + 2)} E_{\alpha + \beta , 1} \bigl( M T^{\alpha + \beta} \bigr) \biggr) \lVert u \rVert \\& \quad = \lVert h \rVert T^{\alpha }E_{\alpha + \beta , \alpha + 1} \bigl( M T^{\alpha + \beta} \bigr) + \biggl[ 3 \bigl\vert f(u) \bigr\vert + \frac{2 \lVert h \rVert T^{\alpha }}{\Gamma (\alpha + 2)} + \frac{2 \vert \lambda \vert }{T} \biggr] E_{\alpha + \beta , 1} \bigl( M T^{ \alpha + \beta} \bigr) < + \infty . \end{aligned}$$

By our assumption,

$$ 1 - \frac{2 M T^{\alpha + \beta}}{\Gamma (\alpha + \beta + 2)} E_{ \alpha + \beta , 1} \bigl( M T^{\alpha + \beta} \bigr) > 0, $$

which infers that $\lVert u \rVert $ is bounded. Furthermore, $u(x)$ is clearly continuous over the interval $[0, T]$. This completes the proof of Lemma 1. □

## Main results

We are now ready to present our key results regarding the uniqueness of solutions to equation (1.1) using Banach’s contractive principle.

### Theorem 2

*Assume that* $a(x) \in C[0, T]$, $g : [0, T] \times R \rightarrow R$ *is continuous and* $f : C[0, T] \rightarrow R$ *is a continuous functional*, *and there exist nonnegative constants* *L* *and* $L_{1}$ *such that*

$$\begin{aligned}& \bigl\vert g (x, y_{1}) - g(x, y_{2}) \bigr\vert \leq L \vert y_{1} - y_{2} \vert , \quad y_{1}, y_{2} \in R, \\& \bigl\vert f(u) - f(v) \bigr\vert \leq L_{1} \lVert u - v \rVert , \quad u, v \in C[0, T]. \end{aligned}$$

*Furthermore*,

$$\begin{aligned} q = & L T^{\alpha }E_{\alpha + \beta , \alpha + 1} \bigl(M T^{ \alpha + \beta } \bigr) + \biggl[3L_{1} + \frac{2 L T^{\alpha}}{\Gamma (\alpha + 2)} + \frac{2 M T^{\alpha + \beta}}{\Gamma (\alpha + \beta + 2)} \biggr] E_{ \alpha + \beta , 1} \bigl(M T^{\alpha + \beta } \bigr) \\ < & 1. \end{aligned}$$

*Then equation* (1.1) *has a unique solution in the space* $C[0, T]$.

### Proof

From Lemma 1, we define a nonlinear mapping *T* over the space $C[0, T]$ by

$$\begin{aligned} (Tu) (x) = & \sum_{j = 0}^{\infty }(-1)^{j} \bigl(I^{\alpha }a(x) I^{\beta} \bigr)^{j} I^{\alpha }g(x, u) - f(u) \sum_{j = 0}^{ \infty }(-1)^{j} \bigl(I^{\alpha }a(x) I^{\beta} \bigr)^{j} \cdot 1 \\ &{}- \frac{2}{\Gamma (\alpha + 1) T^{2}} \int _{0}^{T} ( T - t)^{ \alpha }g\bigl(t, u(t) \bigr) \,dt \cdot \sum_{j = 0}^{\infty }(-1)^{j} \bigl(I^{ \alpha }a(x) I^{\beta} \bigr)^{j} x \\ &{}+ \frac{2}{\Gamma (\alpha + 1) \Gamma (\beta ) T^{2} } \int _{0}^{T} (T - x_{1})^{\alpha }a (x_{1}) \,d x_{1} \int _{0}^{x_{1}} (x_{1} - t)^{ \beta - 1} u(t) \,d t \\ &{}\cdot \sum_{j = 0}^{\infty }(-1)^{j} \bigl(I^{\alpha }a(x) I^{\beta} \bigr)^{j} x + \frac{2 \lambda }{T^{2}} \sum_{j = 0}^{\infty }(-1)^{j} \bigl(I^{\alpha }a(x) I^{\beta} \bigr)^{j} x \\ &{}+ \frac{2 f(u)}{T} \sum_{j = 0}^{\infty }(-1)^{j} \bigl(I^{ \alpha }a(x) I^{\beta} \bigr)^{j} x. \end{aligned}$$

Clearly,

$$\begin{aligned} \bigl\vert g(x, u) \bigr\vert = \bigl\vert g( x, u) - g(x, 0) + g(x, 0) \bigr\vert \leq L \vert u \vert + \bigl\vert g(x, 0) \bigr\vert , \end{aligned}$$

which implies that

$$ \max_{x \in [0, T]} \bigl\vert g(x, u) \bigr\vert \leq L \lVert u \rVert + \max_{x \in [0, T]} \bigl\vert g(x, 0) \bigr\vert < + \infty , $$

since $g(x, 0) \in C[0, T]$. It follows from inequality (1.5) that $(Tu)(x) \in C[0, T]$ by noting that

$$ 1 - \frac{2 M T^{\alpha + \beta}}{\Gamma (\alpha + \beta + 2)} E_{ \alpha + \beta , 1} \bigl( M T^{\alpha + \beta} \bigr) > 0 $$

in Lemma 1, as $q < 1$. Further, we need to prove that *T* is contractive. Indeed,

$$\begin{aligned} &(Tu) (x) - (Tv) (x) \\ &\quad = \sum_{j = 0}^{\infty }(-1)^{j} \bigl(I^{ \alpha }a(x) I^{\beta} \bigr)^{j} I^{\alpha } \bigl( g(x, u) - g(x, v)\bigr) \\ &\qquad {}- \bigl(f(u) - f(v)\bigr) \sum_{j = 0}^{\infty }(-1)^{j} \bigl(I^{\alpha }a(x) I^{\beta} \bigr)^{j} \cdot 1 \\ &\qquad {}- \frac{2}{\Gamma (\alpha + 1) T^{2}} \int _{0}^{T} ( T - t)^{ \alpha }\bigl(g\bigl(t, u(t)\bigr) - g\bigl(t, v(t)\bigr)\bigr) \,dt \cdot \sum _{j = 0}^{\infty }(-1)^{j} \bigl(I^{\alpha }a(x) I^{\beta} \bigr)^{j} x \\ &\qquad {}+ \frac{2}{\Gamma (\alpha + 1) \Gamma (\beta ) T^{2} } \int _{0}^{T} (T - x_{1})^{\alpha }a (x_{1}) \,d x_{1} \int _{0}^{x_{1}} (x_{1} - t)^{ \beta - 1} \bigl(u(t) - v(t)\bigr) \,d t \\ &\qquad {}\cdot \sum_{j = 0}^{\infty }(-1)^{j} \bigl(I^{\alpha }a(x) I^{\beta} \bigr)^{j} x + \frac{2 (f(u)- f(v))}{T} \sum_{j = 0}^{\infty }(-1)^{j} \bigl(I^{\alpha }a(x) I^{\beta} \bigr)^{j} x. \end{aligned}$$

Thus,

$$\begin{aligned} \bigl\vert (Tu) (x) - (Tv) (x) \bigr\vert \leq& L T^{\alpha } \lVert u - v \rVert \sum_{j = 0}^{\infty } \frac{M^{j} T^{(\alpha + \beta ) j }}{\Gamma ((\alpha + \beta ) j + \alpha + 1)} \\ &{}+ 3 L_{1} \lVert u - v \rVert \sum_{j = 0}^{\infty } \frac{M^{j} T^{(\alpha + \beta ) j}}{\Gamma ((\alpha + \beta )j + 1)} \\ &{}+ \frac{2 L \lVert u - v \rVert T^{\alpha}}{\Gamma (\alpha + 2)} \sum_{j = 0}^{\infty } \frac{M^{j} T^{(\alpha + \beta ) j}}{\Gamma ((\alpha + \beta )j + 1)} \\ &{}+ \frac{2 M \lVert u - v \rVert T^{\alpha + \beta}}{\Gamma (\alpha + \beta + 2)} \sum_{j = 0}^{\infty } \frac{M^{j} T^{(\alpha + \beta ) j}}{\Gamma ((\alpha + \beta )j + 1)} \\ =& q \lVert u - v \rVert . \end{aligned}$$

Since $q < 1$, equation (1.1) has a unique solution in the space $C[0, T]$ by Banach’s fixed point theorem. This completes the proof of Theorem 2. □

### Example 3

The following nonlinear integro-differential equation with nonlocal boundary condition and variable coefficients:

$$\begin{aligned} \textstyle\begin{cases} {}_{C} D^{3.5} u(x) + \frac{2 x^{2}}{x^{2} + 1} I^{1.1} u(x) = \frac{1}{2} \cos (x^{2} u(x)) + x^{3}, \quad x \in [0, 1], \\ u(0) = \frac{1}{9} \sin u(1/2), \qquad u''(0) = u'''(0) = 0, \\ \int _{0}^{1} u(x) \,d x = \sqrt{2}, \end{cases}\displaystyle \end{aligned}$$

has a unique solution in $C [0, 1]$.

### Proof

Clearly,

$$ g(x, u) = \frac{1}{2} \cos \bigl(x^{2} u(x)\bigr) + x^{3}, $$

and

$$ \bigl\vert g(x, u) - g(x, v) \bigr\vert \leq \frac{1}{2} \bigl\vert x^{2} u - x^{2} v \bigr\vert \leq \frac{1}{2} \vert u - v \vert . $$

Therefore, $L = 1/2$ as $x \in [0, 1]$. On the other hand,

$$ f(u) = \frac{1}{9} \sin u(1/2), $$

and

$$ \bigl\vert f(u) - f(v) \bigr\vert \leq \frac{1}{9} \bigl\vert \sin u(1/2) - \sin v(1/2) \bigr\vert \leq \frac{1}{9} \bigl\vert u(1/2) - v(1/2) \bigr\vert \leq \frac{1}{9} \lVert u - v \rVert , $$

which indicates that $L_{1} = 1/9$. It follows from Theorem 2 that

$$ q = \frac{1}{2} E_{4, 6, 4.5}(2) + \biggl[\frac{3}{9} + \frac{1}{\Gamma (5.5)} + \frac{4}{\Gamma (6.6)} \biggr] E_{4.6, 1}(2). $$

Using online calculators from the site https://www.wolframalpha.com/ (accessed on 09 October 2022), we get

$$\begin{aligned}& E_{4, 6, 4.5}(2) = \sum_{j = 0}^{\infty } \frac{2^{j}}{\Gamma (4.6 j + 4.5 )} \approx 0.0860118, \\& E_{4.6, 1}(2) = \sum_{j = 0}^{\infty } \frac{2^{j}}{\Gamma (4.6 j + 1 )} \approx 1.0325, \\& \frac{1}{\Gamma (5.5)} \approx 0.0191048, \qquad \frac{4}{\Gamma (6.6)} \approx 0.0116042. \end{aligned}$$

These obviously claim that $q < 1$. By Theorem 2, it has a unique solution in $C [0, 1]$. □

## Conclusion

We have investigated the uniqueness of solutions to the nonlinear fractional integro-differential equation (1.1) with nonlocal boundary condition and variable coefficients using the Mittag-Leffler function, Babenko’s approach, and Banach’s contractive principle and presented an applicable example. The technique used clearly opens up new directions for studying other types of boundary conditions or with different fractional derivatives, such as boundary value problems of the nonlinear fractional partial integro-differential equations with variable coefficients as well as nonlinear integro-differential equations with the Hilfer fractional derivatives.

## Data Availability

No data were used to support this study.
